# Association of Maternal Exposure to Fine Particulate Matter During Pregnancy with Anterior Segment Dysgenesis Risk: A Matched Case-Control Study

**DOI:** 10.3390/jcm14093003

**Published:** 2025-04-26

**Authors:** Sooyeon Choe, Kyung-Shin Lee, Ahnul Ha, Soontae Kim, Jin Wook Jeoung, Ki Ho Park, Yun-Chul Hong, Young Kook Kim

**Affiliations:** 1 Department of Ophthalmology, Chungnam National University College of Medicine, Daejeon 35015, Republic of Korea; 2 Department of Ophthalmology, Chungnam National University Hospital, Daejeon 35015, Republic of Korea; 3 Public Health Research Institute, National Medical Center, Seoul 04564, Republic of Korea; 4 Department of Ophthalmology, Jeju National University Hospital, Jeju 63241, Republic of Korea; 5 Department of Ophthalmology, Jeju National University School of Medicine, Jeju 63243, Republic of Korea; 6 Department of Environmental and Safety Engineering, Ajou University, Suwon 16499, Republic of Korea; 7 Department of Ophthalmology, Seoul National University College of Medicine, Seoul 03080, Republic of Korea; 8 Department of Ophthalmology, Seoul National University Hospital, Seoul 03080, Republic of Korea; 9 Department of Human Systems Medicine, Seoul National University College of Medicine, Seoul 03080, Republic of Korea; 10 Institute of Environmental Medicine, Seoul National University Medical Research Center, Seoul 03080, Republic of Korea; 11 Department of Pediatric Ophthalmology, Seoul National University Children’s Hospital, Seoul 03080, Republic of Korea

**Keywords:** fine particulate matter, anterior segment dysgenesis, congenital anomaly, air pollution, pregnancy

## Abstract

**Background/Objectives:** To assess the association of residential-level maternal particulate matter of 2.5 μm diameter or less (PM_2.5_) exposure during pregnancy with anterior segment dysgenesis (ASD) risk. **Methods:** This study used data from children diagnosed with ASD (i.e., aniridia, iris hypoplasia, Peters anomaly, Axenfeld–Rieger syndrome, or primary congenital glaucoma) by an experienced pediatric ophthalmologist at a National Referral Center for Rare Diseases between 2004 and 2021 and their biological mothers. Individual PM_2.5_ exposure concentration was assessed by reference to residential addresses and district-specific PM_2.5_ concentrations predicted by the universal Kriging prediction model. **Results:** The study included 2328 children (582 ASD cases and 1746 controls [1:3 matched for birth year, sex, and birth-place]). The mean (SD) annual PM_2.5_ exposure was 29.2 (16.9) μg/m^3^. An IQR increase in PM_2.5_ during the preconception period (11.6 μg/m^3^; RR, 1.18; 95% CI, 1.03–1.34), the 1st trimester (11.1 μg/m^3^; RR, 1.15; 95% CI, 1.03–1.27), and the 2nd trimester (11.2 μg/m^3^; RR 1.14; 95% CI, 1.01–1.29) significantly increased ASD risk. Meanwhile, the association between IQR increase in PM_2.5_ during the 3rd trimester and ASD risk showed borderline significance (11.0 μg/m^3^; RR, 1.10; 95% CI, 0.99–1.21). An IQR increase in PM_2.5_ (6.9 μg/m^3^) from the preconception period to the 3rd trimester was associated with a significantly increased risk of ASD (RR, 1.13; 95% CI, 1.08–1.20). **Conclusions:** The findings of this study suggest that PM_2.5_ exposure during the preconception period and pregnancy is associated with increased risk of ASD, supporting a need for further improvements in air quality to prevent congenital ocular anomalies.

## 1. Introduction

Air pollution, fine particulate matter (PM_2.5_, aerodynamic diameter ≤ 2.5 μm) in particular, is among the greatest environmental threats to public health. A growing number of epidemiological studies have found that prenatal exposure to PM_2.5_ is associated with deleterious outcomes in fetuses [1,2]. The indications are that maternal PM_2.5_ exposure is implicated in certain pediatric conditions, among which are congenital heart diseases [3,4], childhood cancer [5,6], and respiratory problems [7,8]. Generally, it is believed that pollutants can act directly on the fetus via the placental barrier, or, more commonly, can induce inflammatory reactions and/or systemic immune responses in the mother, ultimately resulting in adverse outcomes for the child [9].

Anterior segment dysgenesis (ASD) is an umbrella term for eye conditions caused by malformation of the eyeball’s anterior segment structures (i.e., cornea; iris; lens; ciliary body; trabecular meshwork) [10], alterations of which are due to changes (mutations) in one among any of the genes that are involved in normal anterior segment development [11]. Sometimes only one of the anterior segment structures are affected, though more commonly, a number of them are. ASD significantly impacts vision, owing to disruption of the visual axis. Additionally, patients affected by any of the types of ASD have an increased risk of glaucoma and significant visual disability (due to optic nerve damage) resulting therefrom [12].

Previous reports have suggested that PM_2.5_ is implicated in various ocular diseases, such as glaucoma [13,14], ocular surface diseases [15], and retinal diseases (e.g., retinal layer thinning, age-related macular degeneration, and diabetic retinopathy) [16,17,18]. However, little is known about the specific associations between exposure to PM_2.5_ and risk of ASD. Given that congenital heart diseases arising from neural crest cells have been associated with PM_2.5_ exposure, we hypothesized that ocular structures derived from the same cells could also be affected by PM_2.5_ exposure during pregnancy.

The present study aimed to characterize the association of maternal exposure to PM_2.5_ during the preconception period and the 1st, 2nd, and 3rd trimesters of pregnancy with risk of ASD occurrence.

## 2. Methods

### 2.1. Study Design and Participants

Seoul National University Children’s Hospital serves as the National Referral Center for Rare Diseases. In this retrospective, matched case-control study, we included patients identified by the Clinical Data Warehouse (CDW) of Seoul National University Hospital Patients Research Environment (SUPREME). We screened the database for children meeting the following criteria: (1) date of birth between January 2007 and June 2020; (2) having diagnostic codes for anterior segment dysgenesis (i.e., aniridia; Q13.1, iris hypoplasia; Q13.2, Peters anomaly; Q13.4, Axenfeld–Rieger syndrome; Q13.8, primary congenital glaucoma; Q15.0) according to the International Classification of Diseases, 10th revision; (3) confirmation of classification according to diagnosis by an experienced pediatric ophthalmologist in outpatient or inpatient clinic based on electronic medical record and examination findings, and (4) having complete information regarding residential area at the time of or within 6 months of birth, which was used to assign PM_2.5_ exposure.

Data on children who had had hospital visits due to simple refractive errors were used for the control group. The inclusion criteria were (1) date of birth between January 2007 and June 2020; (2) having diagnostic codes for refractive errors (i.e., hyperopia; H52.0, myopia; H52.1, astigmatism; H52.2) without codes for ASD; (3) confirmation of not having other congenital ocular abnormalities by pediatric ophthalmologists in outpatient or inpatient clinic. A greedy method was adopted to select controls by matching according to a 1:3 case: control ratio with adjustment for age, sex, and birth place (city or province) [19].

Baseline sociodemographic data included date of birth, sex, residential area (city or province), and type of insurance. The Institutional Review Board of Seoul National University Hospital approved the study protocol, which adhered to the tenets of the Declaration of Helsinki, and enabled use of de-identified data without individual patient consent.

To estimate individual-level PM_2.5_ exposure, we assigned district-level PM_2.5_ concentrations based on each participant’s maternal residential district at the time of childbirth. Residential information was linked to administrative district codes, which were then matched with modeled and interpolated PM_2.5_ data.

### 2.2. Exposure Time Windows

**Figure 1:**
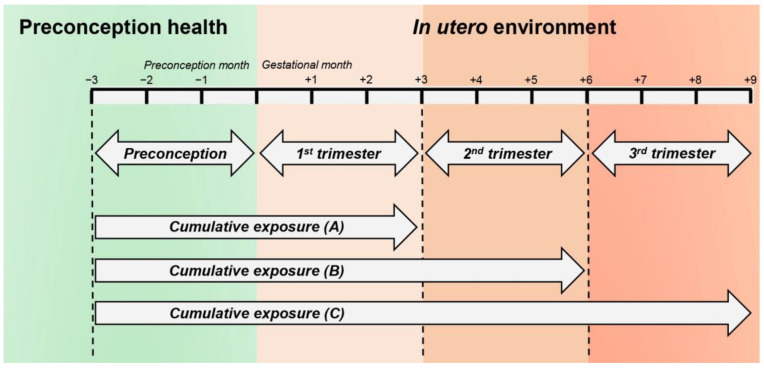
PM_2.5_ exposure assessment periods. (1) Preconception period; (2) From preconception period to 1st trimester; (3) From preconception period to 2nd trimester; (4) From preconception period to 3rd trimester; (5) 1st trimester; (6) 2nd trimester; (7) 3rd trimester.

The exposure window involved in this study covers the two main preconception and prenatal periods. The preconception period was defined as 3 months before conception. The prenatal period was divided into three separate timing windows: 1st trimester (the first three months of pregnancy), 2nd trimester (the fourth through sixth months of pregnancy), and 3rd trimester (the seventh month of pregnancy to birth) of gestation. Additionally, since we had hypothesized that cumulative exposure to PM_2.5_ and ASD risk would be positively correlated, we calculated cumulative exposure from the preconception period to each trimester (Figure 1).

### 2.3. District-Specific Estimation of PM_2.5_ Exposure

The main exposure level of each participant according to their district area of residence in 2006–2020 was PM_2.5_. We principally used observational data and air-quality modeling results to calculate the exposure level of PM_2.5_ during 2006–2015. PM_2.5_ observation in South Korea was officially initiated in 2015 (http://www.airkorea.or.kr/eng [accessed on 3 April 2021]). To derive the nationwide PM_2.5_ concentration field for the years 2006–2014 during which no continuous public PM_2.5_ observations were available, we multiplied modeled PM10 concentrations by the ratio of observed PM_2.5_ to PM10 concentrations from 2015 to 2020. Prior to the application, modeled PM10 concentrations were adjusted based on PM10 observations. Backward projection was adopted to take the annual change in the ratios into consideration. For the years during which PM_2.5_ observations are available, Kriging spatial interpolation of PM_2.5_ observations was applied. Modeling outputs for 2015–2020 were partially utilized to provide PM_2.5_ concentrations for locations where PM_2.5_ observations are unavailable (i.e., islands or coastal areas of the Korean Peninsula) during the spatial interpolation [20]. Previously published studies provided a procedure for generating data on PM_2.5_ as an air pollutant [21,22]. The predictive performance of this approach has been previously evaluated [23]. Model validation studies using the same methodology reported an R^2^ value of 0.73 and an RMSE value of 8.25 μg/m^3^, supporting the robustness of this estimation strategy.

To simulate ambient concentrations of PM_2.5_ in South Korea for the period 2006–2020, we used the Community Multiscale Air Quality (CMAQ version 4.7.1) model, which comprehensively represents the most important processes, including atmospheric chemistry, dynamics, diffusion, and advection [24]. The model had data on 251 administrative areas (regional units smaller than a city or province) in 7 cities and 9 provinces. Daily PM_2.5_ concentrations in each of the 16 regions (i.e., 7 cities + 9 provinces) were calculated by averaging the concentrations in the aforementioned administrative areas. We obtained the average concentration of PM_2.5_ in each district as the individual-level exposure. Anthropogenic emissions were processed using Sparse Matrix Operator Kernel Emission (SMOKE version 3.1) [24], and meteorological inputs were calculated by the Weather Research and Forecasting (WRF version 3.3.1) model [25]. Details on the application of CMAQ simulations can be found in previous reports [20,26].

Average hourly ambient temperature (°C) data were gathered from the Korea Meteorological Administration (KMA). From this hourly data, the monthly average PM_2.5_ and temperature were calculated. We assigned district-level PM_2.5_ to each trimester of pregnancy as a proxy for exposure of pregnant women to fine particulate matter based on their residential district.

### 2.4. Statistical Analyses

ArcGIS (v10.2, ESRI, Redlands, CA, USA) was used to visualize the spatial distribution of PM_2.5_ concentrations and the number of ASD patients in South Korea. A multivariate analysis was performed by applying generalized estimating equations (GEEs) to analyze the associations of the periods of preconception and of each trimester during pregnancy with PM_2.5_ and ASD. Compound symmetry was assumed for the covariance structure because we considered the spatial proximity of residents in the same city or province. We adjusted for the child’s birth year, season at birth, sex, policyholder’s income level (1–10th level), and monthly average temperature at birth. The associations of PM_2.5_ were expressed as a percentage change for each congenital disease with an interquartile range (IQR) increase in the PM_2.5_ concentration during each trimester. We interpreted the relative risk (RR) as an increase in ASD for each IQR increase of PM_2.5_ concentration [27].

All the analyses were conducted using SAS (version 9.4; SAS Institute, Cary, NC, USA) and R 3.5.1 (R Foundation for Statistical Computing, Vienna, Austria). The statistical significance level was set at *p*-value < 0.05 (two-tailed). 

## 3. Results

### 3.1. Demographic and Clinical Characteristics of Study Population

After the 1:3 case-control matching process, a total of 582 children (260 [44.7%] girls) with ASD and 1746 children (788 [45.1%] girls) without any congenital ocular abnormalities (the controls) were included in the study. Of the total subjects (2328 children), 1123 (48.2%) resided in cities and 1205 (51.8%) resided in provinces. Among the ASD patients analyzed, 250 (43%) had Axenfeld–Rieger syndrome, 192 (33%) had primary congenital glaucoma, 67 (12%) had Peters anomaly, 65 (11%) had aniridia, and 19 (3%) had iris hypoplasia. Eight patients had both Axenfeld–Rieger syndrome and iris hypoplasia, and three had both Peters anomaly and iris hypoplasia. The detailed baseline characteristics of the study population are provided in Table 1.

### 3.2. PM_2.5_ Exposure in Study Subjects

**Figure 2:**
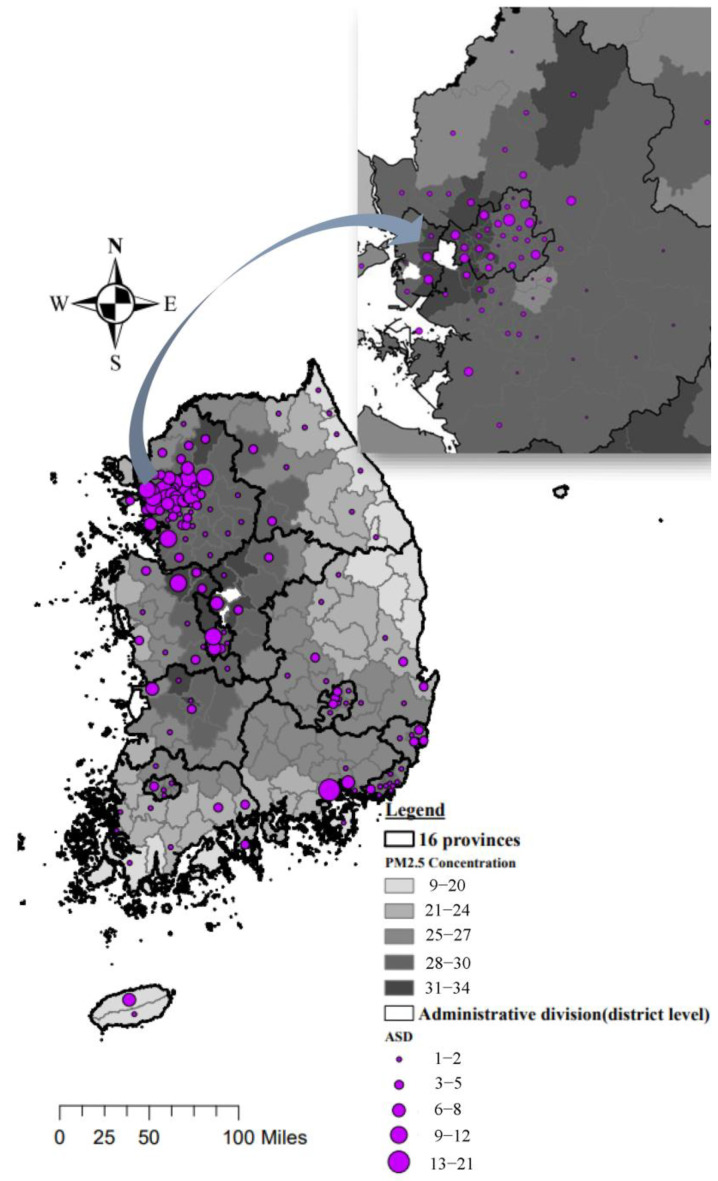
Spatial distributions of PM_2.5_ exposure and number of anterior segment dysgenesis (ASD) patients in South Korea.

The annual mean PM_2.5_ concentrations for the years 2006 through 2020 in each region are presented in Table S1. The average (standard deviation) PM_2.5_ concentration during this 15-year period was 29.2 (16.9) μg/m^3^. The spatial distribution of the number of ASD cases and the average PM_2.5_ exposure concentration for each administrative division is depicted in Figure 2. The PM_2.5_ exposure levels of the ASD patients and control participants during the preconception and prenatal periods are shown in Table 2.

Each administrative division is shaded according to the average PM_2.5_ concentration during the first trimester of pregnancy. Bubble size represents the number of ASD cases within each division, with larger bubbles indicating a greater number of cases. The map scale reflects administrative-level divisions across the national territory. Color intensity corresponds to relative PM_2.5_ exposure levels, with darker shades indicating higher concentrations.

### 3.3. Association Between Maternal PM_2.5_ Exposure and Risk of ASD

Maternal exposure to PM_2.5_ during the preconception period and the 1st and 2nd trimesters was significantly associated with ASD risk. The adjusted RR per IQR (i.e., preconception period: 11.6 μg/m^3^, 1st trimester: 11.1 μg/m^3^, 2nd trimester: 11.2 μg/m^3^) was 1.18 (95% CI, 1.03–1.34), 1.15 (95% CI, 1.03–1.27), and 1.14 (95% CI, 1.01–1.29), respectively. Meanwhile, the association between PM_2.5_ exposure during the 3rd trimester and ASD risk showed borderline significance (RR, 1.10; 95% CI, 0.99–1.21) per IQR increase (11.0 μg/m^3^).

Cumulative PM_2.5_ exposure from the preconception period to the 1st trimester increased ASD risk by 1.16 (95% CI, 1.08–1.26) per IQR increase (8.8 μg/m^3^), that from the preconception period to the 2nd trimester by 1.14 (95% CI, 1.07–1.21) per IQR increase (7.4 μg/m^3^), and that from the preconception period to the 3rd trimester by 1.13 (95% CI, 1.08–1.20) per IQR increase (6.9 μg/m^3^). The full statistical results summary can be viewed in Table 3.

## 4. Discussion

Our matched study, to the best of our knowledge, is the first to find an association between maternal exposure to fine particulate matter and ASD risk in children. The significant association was observed for PM_2.5_ exposure during the three months prior to conception as well as during the 1st and 2nd trimesters. Not surprisingly, cumulative PM_2.5_ exposure during the preconception and prenatal periods further increases ASD risk.

The ocular structures of the anterior segment begin developing at the seventh gestational week [28]. In the eighth week, corneal differentiation continues, and the iris emerges [29]. Later, during the gestational fourth month, differentiation of the trabecular meshwork proceeds [30]. Disruption of these tissues’ development may lead to any of a variety of clinical presentations that are grouped under the ASD rubric. Our findings indicate that PM_2.5_ exposure increases the risk of ASD when it occurs during any of these critical developmental stages, with the exception of exposure limited to the third trimester, which showed only borderline significance. This borderline association during the third trimester should be interpreted with caution. Earlier developmental windows are considered critical periods of ocular organogenesis, during which the fetus may be more susceptible to environmental insults. In contrast, the third trimester represents a later stage of eye development, when structural differentiation is largely complete and thus potentially less vulnerable to toxic exposures. Interestingly, exposure to PM_2.5_ in the period of preconception has also been shown to be associated with ASD risk. In this light, the report of Stephenson et al. [31] emphasized the potential significance of maternal health change in the preconception period.

Some of the possible mechanisms of the association of PM_2.5_ and ASD risk are oxidative stress, inflammation, and gene-expression changes [32,33]. PM_2.5_ exposure reduces antioxidant enzymes and increases the levels of nitric oxide dose-dependently [34], thereby inducing oxidative stress. Excessive amounts of reactive oxygen species (ROS) can disrupt cellular signaling by damaging DNA or protein in cells. Maternal PM_2.5_ exposure has been implicated in placental inflammation via the systemic inflammatory response, which syndrome threatens fetal development [35,36]. PM_2.5_ can also cause DNA and cell spindle damage [37] or induce mutations by inhibition of DNA repair [38]. Beyond these general mechanisms, specific toxic components of PM_2.5_ exert distinct effects. Heavy metals such as lead, cadmium, and arsenic increase ROS generation and interfere with antioxidant defenses, disrupting neural crest cell migration—a critical process in anterior segment formation [39,40]. Polycyclic aromatic hydrocarbons (PAHs) impair DNA repair and promote mutagenesis, potentially affecting genes involved in ocular development [41]. Organic compounds have been shown to induce apoptosis and interfere with vascular formation [42]. These component-specific toxicities provide biologically plausible pathways through which PM_2.5_ may contribute to ASD, highlighting the importance of further research on its composition and fetal ocular effects.

We explored the risk of PM_2.5_ exposure in our two largest diagnosis subgroups (250 Axenfeld–Rieger syndrome patients; 192 primary congenital glaucoma patients). In former patients, higher PM_2.5_ exposure in the preconception period and from the preconception period to each trimester increased the risk of Axenfeld–Rieger syndrome (all *p*-values < 0.05, Table S2); in the latter patients, PM_2.5_ exposure in the 1st trimester was associated with increased risk of primary congenital glaucoma (*p*-value = 0.04, Table S3), and cumulative exposure from preconception to each trimester also showed borderline significance (Table S3). Although these phenotypic subgroups consisted of relatively small numbers of patients, we found a positive association between cumulative PM_2.5_ exposure and each of them. Axenfeld–Rieger syndrome and primary congenital glaucoma share some causative genes (FOXC1 and CYP1B1) that may also induce other subtypes of ASD (aniridia, iris hypoplasia, and Peters anomaly), while the LTBP2 gene is confined to primary congenital glaucoma [10]. Thus, further study with larger sample sizes of other phenotypes may supplement the findings of the current study.

We further explored potential effect modification by including interaction terms for sex × PM_2.5_ and residential area (city vs. province) × PM_2.5_ (Table S4). The results showed significant interaction effects only during the cumulative exposure period from the preconception to the first trimester (sex × PM_2.5_: *p* = 0.045; residential area × PM_2.5_: *p* = 0.038). No statistically significant interaction was observed during the other exposure windows. These findings suggest that the association between PM_2.5_ exposure and ASD risk may differ slightly by sex and residential environment, particularly during early developmental periods. However, further large-scale studies are warranted to confirm the possibility of sex- and environment-specific susceptibility.

The current study has several strengths. First, the ASD diagnoses were confirmed by an experienced pediatric ophthalmologist. Since ASD presents within a diverse spectrum, diagnosis codes may prove insufficiently reliable. Thus, we accessed and reviewed the electronic medical records and examination findings to reconfirm the diagnoses. Second, this study was conducted from a nationwide referral center for child patients with rare diseases in South Korea. As such, its sample size is significant, despite the relative rarity of ASD, and the data may be considered to be representative of South Korea. Lastly, the database contained a wider range of mean PM_2.5_ concentrations by observation district than those utilized in previous studies [43,44] evaluating the influence of PM_2.5_ and other systemic diseases. Hence, our database could be seen to be more appropriate for an investigation of the precise association of exposure to PM_2.5_ and ASD risk.

There are several limitations to this study that should be taken into account when interpreting its results. First, we focused on PM_2.5_ as an overall indicator of air pollution. However, we did not assess its specific chemical components or sources, such as elemental carbon, organic compounds, or heavy metals, which may exert distinct biological effects on fetal ocular development. Moreover, PM_2.5_ may co-occur with other pollutants, such as nitrogen dioxide (NO_2_), ozone, or carbon monoxide, which were not included in our analysis. These co-pollutants may confound or interact with PM_2.5_ exposure. Future studies should consider multi-pollutant models to better delineate their respective contributions to ASD risk. Although our dataset included a wide range of PM_2.5_ concentrations across regions, which allowed for informative dose–response analysis, this strength also warrants cautious interpretation, as regional heterogeneity in exposure measurement and unmeasured spatial confounders may still affect the robustness of our findings. Furthermore, unmeasured social determinants of health—such as maternal educational status, occupational exposures, or access to prenatal care—may also contribute to the observed associations and should be explored in future investigations. Second, the pollutant-level estimations were based on home address at birth. This approach does not account for residential mobility during pregnancy, which could lead to exposure misclassification. Additionally, our estimates captured only outdoor PM_2.5_ concentrations near the home and did not include non-residential environments, such as workplaces, commuting routes, or indoor sources of pollution. Data on second-hand smoke exposure were also not available. However, error in exposure measurement would not be expected to increase ASD risk; any resulting bias, therefore, would be expected to fall towards the null, thereby probably attenuating the effects estimated. Third, whereas the models had been adjusted for any possible confounders known, residual confounding may yet have occurred. We did not control for genetic factors or family history that might predispose to ASD. This may have resulted in residual confounding. Moreover, due to the absence of genetic information, we were unable to evaluate potential gene–environment interactions, which may have contributed to ASD risk. Fourth, we investigated ASD as a group of disorders of the anterior segment of the eye. Since the phenotypes of ASD subgroups (i.e., Axenfeld–Rieger anomaly, aniridia, iris hypoplasia, Peters anomaly, primary congenital glaucoma) overlap with each other, it was impossible to separate them with discrete criteria. Additionally, although we performed subgroup analyses for the two most prevalent ASD types—Axenfeld–Rieger syndrome and primary congenital glaucoma—other subtypes had sample sizes too small for independent statistical analysis. This, combined with phenotypic overlap, limited our ability to conduct subtype-specific investigations. Further studies incorporating genotypes of ASD would be needed to provide a greater pool of information on the association of ASD and PM_2.5_. Additionally, given that ocular ASD is induced by complex correlations of embryological as well as genetic factors [45], future investigations into genetic versus environmental factor interactions would be advised to clarify the role of PM_2.5_ in the development of ASD. Lastly, due to the rarity of anterior segment dysgenesis, we included all eligible cases identified within the national referral center cohort during the study period, thereby ensuring adequate statistical power even though no formal sample size calculation was performed.

In conclusion, we found that maternal exposure to PM_2.5_ during the preconception period and the 1st and 2nd trimesters was significantly associated with development of ASD. This finding suggests that PM_2.5_ is a novel environmental risk factor for ASD that could be minimized by specific measures and with extra caution.

## Figures and Tables

**Table 1:** Demographic characteristics of study participants.

Variable	Patients with ASD	Control Participants *	*p*-Value **
Total	582 (25.0)	1746 (75.0)	
Sex			0.85
Boys	322 (55.3)	958 (54.9)	
Girls	260 (44.7)	788 (45.1)	
Type of insurance			0.07
Medical aid	18 (3.1)	32 (1.8)	
Health insurance	564 (96.9)	1714 (98.2)	
Season at conception date			0.47
Spring	149 (25.6)	490 (28.1)	
Summer	169 (29.0)	459 (26.3)	
Fall	146 (25.1)	425 (24.3)	
Winter	118 (20.3)	372 (21.3)	
Year of birth			1.00
2007	43 (7.4)	122 (7.0)	
2008	29 (5.0)	95 (5.4)	
2009	33 (5.7)	95 (5.4)	
2010	58 (10.0)	174 (10.0)	
2011	47 (8.1)	147 (8.4)	
2012	48 (8.3)	146 (8.4)	
2013	56 (9.6)	163 (9.3)	
2014	57 (9.8)	172 (9.9)	
2015	51 (8.8)	159 (9.1)	
2016	56 (9.6)	163 (9.3)	
2017	31 (5.3)	90 (5.2)	
2018	29 (5.0)	76 (4.4)	
2019	31 (5.3)	101 (5.8)	
2020	13 (2.2)	43 (2.5)	
Cities or Provinces			1.00
City	281 (48.3%)	842 (48.2%)	
Province	301 (51.7%)	904 (51.8%)	

Note: ASD, anterior segment dysgenesis; * Data are presented as number (percentage) of participants; ** *p*-values are calculated using the Chi square test statistic.

**Table 2:** PM_2.5_ exposure during preconception and pregnancy periods in case-control study.

	Period of PM_2.5_ Exposure (μg/m^3^)	Mean	SD	Median	IQR
**Patients with ASD**	Preconception	29.0	8.1	29.3	22.6, 34.7
	1st trimester	28.2	8.0	27.6	22.7, 33.3
	2nd trimester	28.4	7.9	28.1	22.6, 33.8
	3rd trimester	28.7	7.6	28.7	23.3, 33.4
	Preconception—1st trimester	28.6	6.7	28.3	24.0, 32.7
	Preconception—2nd trimester	28.5	5.6	27.9	24.6, 32.3
	Preconception—3rd trimester	28.6	5.2	28.3	25.2, 32.1
**Control Participants**	Preconception	28.7	8.1	28.4	22.7, 34.1
	1st trimester	28.0	7.9	27.8	22.2, 33.4
	2nd trimester	28.0	8.0	27.8	22.0, 33.2
	3rd trimester	28.4	7.8	28.0	22.6, 34.0
	Preconception—1st trimester	28.3	6.6	28.2	24.0, 32.8
	Preconception—2nd trimester	28.2	5.6	28.0	24.6, 31.8
	Preconception—3rd trimester	28.3	5.4	28.0	24.9, 31.9

Note: ASD, anterior segment dysgenesis; PM_2.5_, fine particulate matter measuring 2.5 μm or less; SD, standard deviation; IQR, interquartile range.

**Table 3:** Relative risk (RR) of anterior segment dysgenesis (ASD) per interquartile range (IQR) * increases with PM_2.5_.

Period of PM_2.5_ Exposure	Crude Model		Adjusted Model **	
	RR (95% CI)	*p*-Value	RR (95% CI)	*p*-Value
Preconception	1.05 (0.92, 1.20)	0.470	1.18 (1.03, 1.34)	0.014
1st trimester	1.04 (0.91, 1.18)	0.592	1.15 (1.03, 1.27)	0.009
2nd trimester	1.09 (0.95, 1.24)	0.208	1.14 (1.01, 1.29)	0.037
3rd trimester	1.05 (0.92, 1.20)	0.483	1.10 (0.99, 1.21)	0.061
Preconception—1st trimester	1.06 (0.93, 1.20)	0.376	1.16 (1.08, 1.26)	<0.001
Preconception—2nd trimester	1.08 (0.96, 1.22)	0.220	1.14 (1.07, 1.21)	<0.001
Preconception—3rd trimester	1.07 (0.95, 1.21)	0.254	1.13 (1.08, 1.20)	<0.001

* IQR for the preconception period: 11.6 μg/m^3^. IQR for the first trimester: 11.1 μg/m^3^. IQR for the second trimester: 11.2 μg/m^3^. IQR for the third trimester: 11.0 μg/m^3^. IQR for the period of preconception to first trimester: 8.8 μg/m^3^. IQR for the period of preconception to second trimester: 7.4 μg/m^3^. IQR for the period of preconception to third trimester: 6.9 μg/m^3^. ** The model was adjusted for child’s birth year, sex, season at conception, monthly average temperature at exposure period, residential area (state and city), and type of insurance. Note: PM_2.5_, fine particulate matter measuring 2.5 μm or less; RR, relative risk; CI, confidence interval.

## Data Availability

Dataset available on request from the corresponding authors.

## Supplementary Materials

The following supporting information can be downloaded at: https://www.mdpi.com/article/10.3390/jcm14093003/s1, Table S1. Annual Mean Concentration of PM_2.5_ (μg/m^3^) in 16 Regions (7 Metropolitan cities and 9 Provinces) in South Korea (2006–2020); Table S2. Relative risk (RR) of Axenfeld–Rieger syndrome per interquartile range (IQR) increases with PM_2.5_; Table S3. Relative risk (RR) of primary congenital glaucoma per interquartile range (IQR) increases with PM_2.5_; Table S4. Relative risk (RR) of anterior segment dysgenesis (ASD) per interquartile range (IQR) increase in PM_2.5_ concentration, with interaction terms for sex and residential area.

## Abbreviations

The following abbreviations are used in this manuscript:

ASD	anterior segment dysgenesis
IQR	interquartile range
PM_2.5_	fine particulate matter measuring 2.5 μm or less
RR	relative risk
