# Supplementary material for: Association of Maternal Exposure to Fine Particulate Matter During Pregnancy with Anterior Segment Dysgenesis Risk: A Matched Case-Control Study
Source: J Clin Med. 2025 Apr 26;14(9):3003. doi: 10.3390/jcm14093003 (PMC12072335; doi:10.3390/jcm14093003)
Supplement: Supplementary file 1 [file jcm-14-03003-s001.zip › jcm-3583224-supplementary.pdf]

Supplementary Materials

Association of Maternal Exposure to Fine Particulate Matter during Pregnancy with Anterior Segment Dysgenesis Risk

Table S1. Annual Mean Concentration of $PM_{2.5}$ (μg/m^3) in 16 Regions (7 Metropolitan cities and 9 Provinces) in South Korea (2006-2020)

Table S2. Relative risk (RR) of Axenfeld-Rieger syndrome per interquartile range (IQR) increases with $PM_{2.5}$

Table S3. Relative risk (RR) of primary congenital glaucoma per interquartile range (IQR) increases with $PM_{2.5}$

Table S4. Relative risk (RR) of anterior segment dysgenesis (ASD) per interquartile range (IQR) increase in $PM_{2.5}$ concentration, with interaction terms for sex and residential area

Table S1. Annual Mean Concentration of $PM_{2.5}$ (μg/m^3) in 16 Regions (7 Metropolitan cities and 9 Provinces) in South Korea (2006-2020)

Metropolitan cities

Year	Seoul	Incheon	Daejeon	Daegu	Ulsan	Gwangju	Busan
2006	38.0±32.5	38.3±27.2	33.2±25.6	30.9±19.2	27.9±20.6	33.5±19.1	30.1±17.4
2007	38.5±24.1	37.6±23.9	33.9±26.5	30.2±26.5	28.1±29.0	29.3±22.9	29.3±23.3
2008	34.8±18.7	32.5±17.7	30.4±17.7	32.0±19.7	28.3±16.8	29.6±17.7	25.8±15.5
2009	35.3±20.7	33.1±20.8	29.5±17.5	26.5±13.9	25.7±13.2	27.8±14.8	25.6±12.6
2010	32.7±18.9	30.8±18.7	30.0±18.9	28.5±17.7	25.6±15.5	25.9±19.9	25.4±14.0
2011	30.8±19.4	29.7±18.0	29.6±18.9	26.3±14.6	25.2±16.7	24.2±15.5	25.7±15.3
2012	27.1±13.3	26.2±13.0	26.2±13.3	23.8±10.4	24.2±10.5	21.6±10.1	23.9±9.6
2013	31.8±18.3	27.2±15.8	31.9±19.7	26.9±16.0	28.1±15.0	26.9±17.2	24.5±13.6
2014	32.1±20.1	28.8±18.1	27.6±19.1	24.9±15.4	25.1±16.5	19.7±12.6	24.1±14.8
2015	24.2±12.5	27.6±15.2	27.3±15.3	24.9±11.9	24.6±12.2	24.1±12.9	25.9±12.1
2016	28.0±13.3	26.7±15.2	34.3±16.9	27.9±12.5	24.1±11.4	24.7±12.9	32.8±14.0
2017	24.6±14.5	24.9±14.0	20.9±11.1	23.0±12.3	24.8±12.8	23.9±13.8	25.5±12.2
2018	22.7±15.7	21.8±13.7	21.5±13.2	22.2±13.3	23.0±13.6	24.2±14.6	22.9±12.8
2019	24.9±18.4	23.7±16.2	22.0±15.7	22.6±13.1	20.2±12.0	23.7±16.2	21.4±11.5
2020[a]	23.9±11.6	20.3±10.6	20.1±9.0	21.9±10.2	18.0±8.3	19.4±9.8	17.9±8.7

Provinces

Year	GW	GG	CB	CN	GB	GN	JB	JN	JJ
2006	22.3±18.1	35.6±28.0	31.0±22.9	30.9±23.9	24.0±16.3	26.0±14.8	30.3±17.9	24.0±13.7	12.3±7.0
2007	22.5±17.0	35.3±21.3	33.6±21.4	32.1±20.8	24.8±21.0	26.5±23.5	31.4±21.1	23.6±19.0	11.0±8.0
2008	23.1±13.4	31.6±16.7	31.4±16.1	28.6±14.6	23.7±14.0	25.7±15.2	28.1±15.5	23.6±15.3	11.2±8.2
2009	22.7±14.3	31.9±18.8	30.5±18.3	28.1±16.7	21.4±12.1	24.7±12.3	27.8±15.3	23.1±12.9	11.0±7.0
2010	22.0±14.0	30.6±17.5	31.2±20.1	27.8±17.8	21.4±15.2	23.9±14.7	27.9±16.9	21.1±15.5	12.4±12.3
2011	21.7±13.6	29.5±17.2	30.1±17.7	26.5±16.9	21.2±13.1	23.8±14.9	27.1±15.7	20.3±13.3	11.0±8.4
2012	19.7±11.3	25.7±12.6	27.7±13.9	24.6±13.0	20.1±10.0	21.4±9.2	24.9±12.1	18.5±9.6	9.3±5.6
2013	21.8±14.0	28.3±16.1	31.6±20.2	27.1±17.4	21.9±13.3	25.0±14.0	27.6±17.1	19.6±13.2	9.4±7.8
2014	19.5±13.8	28.6±17.5	28.3±20.0	24.0±16.1	20.5±13.7	23.1±14.3	24.0±16.0	17.0±12.0	10.2±10.0
2015	20.6±12.6	25.0±13.8	26.5±14.8	25.8±14.9	21.6±12.3	25.0±11.6	29.1±16.6	22.5±12.3	13.6±10.0
2016	29.3±12.0	30.9±14.3	34.3±15.6	31.4±16.2	26.6±13.3	27.1±12.6	31.4±15.9	23.8±12.4	21.8±12.5
2017	24.6±14.5	26.9±17.1	26.0±16.2	23.0±13.1	23.3±13.4	23.0±10.6	27.6±15.6	20.6±9.7	21.0±10.1
2018	21.3±13.1	24.9±17.7	25.0±16.1	21.0±14.3	23.8±14.7	19.7±11.4	23.7±15.5	21.1±12.9	19.0±9.9
2019	20.1±14.1	25.4±19.7	27.9±19.1	25.4±18.3	20.4±13.8	18.3±10.3	25.0±18.1	19.9±14.3	19.0±11.2

**2020[a]**	19.8±10.7	24.2±12.7	25.3±12.5	24.7±13.0	18.7±9.6	16.9±8.1	22.9±11.7	17.6±9.7	17.7±8.9

Data are shown as mean ± standard deviation.
[a]Average of values from January to June.
$PM_{2.5}$, fine particulate matter measuring 2.5 μm or less; GW, Gangwon-do; GG, Gyeonggi-do; CB, Chungcheongbuk-do; CN, Chungcheongnam-do; GB, Gyeongsangbuk-do; GN, Gyeongsangnam-do; JB, Jeollabuk-do; JN, Jeollanam-do; JJ, Jeju-do

Table S2. Relative risk (RR) of Axenfeld-Rieger syndrome per interquartile range (IQR)[a] increases with $PM_{2.5}$

Period of $PM_{2.5}$ exposure	Crude model		Adjusted model[b]	
	RR (95% CI)	*P* Value	RR (95% CI)	*P* Value
Preconception	1.01 (0.82 , 1.24)	0.922	**1.16 (1.03 , 1.32)**	**0.015**
1st trimester	0.99 (0.81 , 1.22)	0.948	1.13 (0.99 , 1.29)	0.075
2nd trimester	1.11 (0.90 , 1.37)	0.329	1.08 (0.91 , 1.30)	0.376
3rd trimester	1.15 (0.94 , 1.41)	0.173	1.16 (0.99 , 1.35)	0.071
Preconception – 1st trimester	1.00 (0.83 , 1.22)	0.962	**1.14 (1.03 , 1.27)**	**0.015**
Preconception – 2nd trimester	1.04 (0.86 , 1.26)	0.681	**1.11 (1.02 , 1.20)**	**0.017**
Preconception – 3rd trimester	1.08 (0.89 , 1.32)	0.435	**1.12 (1.04 , 1.21)**	**0.004**

[a]IQR for the preconception period: 11.3 μg/m^3. IQR for the first trimester: 11.5 μg/m^3. IQR for the second trimester: 11.4 μg/m^3. IQR for the third trimester: 10.6 μg/m^3. IQR for the period of preconception to first trimester: 8.8 μg/m^3. IQR for the period of preconception to second trimester: 7.4 μg/m^3. IQR for the period of preconception to third trimester: 7.1 μg/m^3. [b]The model was adjusted for child's birth year, sex, season at conception, monthly average temperature at exposure period, residential area (state and city), and type of insurance.

$PM_{2.5}$, fine particulate matter measuring 2.5 μm or less; RR, relative risk; CI, confidence interval

Table S3. Relative risk (RR) of primary congenital glaucoma per interquartile range (IQR)[a] increases with $PM_{2.5}$

Period of $PM_{2.5}$ exposure	Crude model		Adjusted model[b]	
	RR (95% CI)	*P* Value	RR (95% CI)	*P* Value
Preconception	1.14 (0.91 , 1.43)	0.265	1.22 (0.85 , 1.75)	0.280
1st trimester	1.03 (0.82 , 1.30)	0.779	**1.21 (1.01 , 1.46)**	**0.041**
2nd trimester	1.05 (0.84 , 1.33)	0.658	1.17 (0.91 , 1.50)	0.235
3rd trimester	1.00 (0.79 , 1.27)	0.986	0.99 (0.80 , 1.21)	0.890
Preconception – 1st trimester	1.11 (0.89 , 1.39)	0.357	1.21 (0.98 , 1.49)	0.081
Preconception – 2nd trimester	1.10 (0.89 , 1.37)	0.382	1.16 (0.99 , 1.37)	0.061
Preconception – 3rd trimester	1.07 (0.87 , 1.32)	0.517	1.12 (0.99 , 1.27)	0.057

[a]IQR for the preconception period: 11.2 μg/m^3. IQR for the first trimester: 10.9 μg/m^3. IQR for the second trimester: 11.1 μg/m^3. IQR for the third trimester: 11.3 μg/m^3. IQR for the period of preconception to first trimester: 8.8 μg/m^3. IQR for the period of preconception to second trimester: 7.5 μg/m^3. IQR for the period of preconception to third trimester: 6.9 μg/m^3. [b]The model was adjusted for child's birth year, sex, season at conception, monthly average temperature at exposure period, residential area (state and city), and type of insurance.

$PM_{2.5}$, fine particulate matter measuring 2.5 μm or less; RR, relative risk; CI, confidence interval

Table S4. Relative risk (RR) of anterior segment dysgenesis (ASD) per interquartile range (IQR)[a] increase in $PM_{2.5}$ concentration, with interaction terms for sex and residential area

Period of $PM_{2.5}$ exposure	Sex × $PM_{2.5}$ Interaction[b,c]		Residential Area × $PM_{2.5}$ Interaction[b,d]	
	RR (95% CI)	*P* Value	RR (95% CI)	*P* Value
Preconception	1.05 (0.98–1.13)	0.12	1.05 (0.98–1.12)	0.09
1st trimester	1.04 (0.97–1.12)	0.18	1.04 (0.97–1.10)	0.13
2nd trimester	1.03 (0.96–1.10)	0.22	1.03 (0.96–1.09)	0.19
3rd trimester	1.02 (0.94–1.11)	0.31	1.02 (0.95–1.08)	0.28
Preconception – 1st trimester	1.06 (1.00–1.13)	0.045	1.06 (1.00–1.12)	0.038
Preconception – 2nd trimester	1.05 (0.99–1.11)	0.061	1.05 (0.99–1.10)	0.052
Preconception – 3rd trimester	1.04 (0.98–1.10)	0.075	1.04 (0.98–1.09)	0.067

[a]IQR for the preconception period: 11.2 μg/m^3. IQR for the first trimester: 10.9 μg/m^3. IQR for the second trimester: 11.1 μg/m^3. IQR for the third trimester: 11.3 μg/m^3. IQR for the period of preconception to first trimester: 8.8 μg/m^3. IQR for the period of preconception to second trimester: 7.5 μg/m^3. IQR for the period of preconception to third trimester: 6.9 μg/m^3. [b]The model was adjusted for child's birth year, sex, season at conception, monthly average temperature at exposure period, residential area (state and city), and type of insurance (when not included as an interaction term). [c]For the sex interaction term, male was treated as the reference category. [d]For the residential area interaction term, province was treated as the reference category.

$PM_{2.5}$, fine particulate matter measuring 2.5 μm or less; RR, relative risk; CI, confidence interval
